# Should we consider Systemic Inflammatory Response Index (SIRI) as a new diagnostic marker for rectal cancer?

**DOI:** 10.1007/s12672-024-00895-4

**Published:** 2024-02-21

**Authors:** Hilmi Yazici, Ayse Eren Kayaci, Halil Ibrahim Sevindi, Wafi Attaallah

**Affiliations:** Marmara Universitesi Pendik Eğitim Ve Araştırma Hastanesi, Istanbul, Turkey

**Keywords:** Inflammation, Rectal surgery, Overall, Survival, Complication

## Abstract

**Purpose:**

The Systemic Inflammatory Response Index (SIRI), which depends on peripheral neutrophil, monocyte, and lymphocyte count, was found to be an effective prognostic indicator for various malignancies. In this study, we aimed to investigate the diagnostic value and the prognostic impact of SIRI on rectal cancer patients.

**Method:**

The medical records of patients underwent sphincter-sparing rectal cancer surgery at general surgery between 2017 and 2022 were examined retrospectively. Patient demographics, operation types, neoadjuvant chemo/radiotherapies, pathological results, and complications were recorded. A total number of 99 patients who operated with diagnoses other than cancer were conducted as a control group. SIRI was calculated from preoperative peripheral blood samples’ neutrophil, lymphocyte, and monocyte count. The optimal cut-off value for SIRI was found to be 1.38. The clinicopathological outcomes and Overall Survival (OS) were analyzed under two groups according to the SIRI values lower or higher than 1.38.

**Results:**

The number of eligible patients was 104. The median age of the entire cohort was 62 (31–89). The median follow-up time was 33 (1–62) months. The median SIRI value in the study group was significantly higher compared with the control group. The study group was examined under two groups: SIRI 1.38 and SIRI > 1.38. The male gender was significantly more frequent in the high SIRI group. The remaining patient demographics and operation types were similar between the groups. The pathological outcomes were similar between the two groups. Overall Survival rate was better in the low SIRI group than those higher. The higher group had significantly higher complication rates than the lower SIRI group (p: 0.004).

**Conclusion:**

SIRI may be a valuable diagnostic marker in rectal cancer patients. Higher SIRI levels were also associated with poorer prognosis and increased complication rates. Still, further prospective studies with a larger number of patients are needed.

## Introduction

Colorectal cancer (CRC) is the second most common cancer and the second leading cause of cancer-related death worldwide [1, 2]. Over the past 20 years, many prognostic factors, such as cancer stage, lymph node ratio, biological markers, and genetic subtypes (K-ras and BRAF mutations and microsatellite instability), have been linked to CRC prognosis [3, 4]. Furthermore, recently applied surgical treatment modalities such as total mesorectal excision (TME) and radiotherapy have been approved for a significant prognostic effect in the treatment of rectal cancers [5, 6]. Moreover, the recently emerged new chemotherapy agents, immunotherapy, and targeted therapy also changed the prognosis of the disease [7, 8]. However, further prognostic factors have been evaluated in the last decade. The association between the Neutrophil to Lymphocyte Ratio (NLR), Monocyte to Lymphocyte Ratio (MLR), and Platelet to Lymphocyte Ratio (PLR) and the prognosis of various malignancies, including colorectal cancers, has been evaluated in numerous studies [9–12]. Recently, the systemic inflammation response index (SIRI), which is based on the numbers of neutrophils, monocytes, and lymphocytes in peripheral blood (neutrophil x monocyte/lymphocyte), has emerged to be a new prognostic indicator for several types of malignancies [13, 14]. In addition, previous studies were mainly focused on SIRI’s diagnostic impact on infectious conditions. However, no studies have been conducted to determine the diagnostic significance of SIRI in cancer patients, recently. Especially in the last decades, Rectal Cancers were significantly separated from the remaining Colonic Cancers. In the main guidelines, treatment modalities are different between these two groups. Colon cancers show different specific behaviors related to their location in the colon. Therefore, in this study, we aimed to evaluate only Rectal Cancer patients to obtain a more homogeneous study group. Thus, in this study, we evaluated whether SIRI can be used as a new diagnostic marker in rectal cancer.

In this study, we aimed to investigate whether SIRI can be used as a new diagnostic marker in rectal cancer.

## Material-method

This study was designed as a case–control retrospective study. The study group was conducted on patients with rectal cancer who underwent elective sphincter-sparing rectal surgery (SSRS) in Marmara University Hospital between January 2017 and December 2022. The control group was composed of patients who underwent elective surgery for reasons other than cancer at the same interval in a single center. To investigate the prognostic value of SIRI on postoperative complications in SSRS, we excluded the patients who underwent abdominoperineal resection (APR), underwent SSRS with a temporary diverting stoma, underwent a Hartmann’s Procedure, and underwent emergent surgery. The control group was conducted with patients who were operated on during the same period because of other than cancer. In the control group, patients who have a history of previous cancer diagnosis were not enrolled in the study. To remove confusion related to infections, patients with abnormal white blood cell (WBC) counts before surgery were also excluded from both groups. Patient demographics, preoperative laboratory tests, surgical procedures, postoperative complications according to Clavien-Dindo, histopathological reports, received neo/adjuvant treatment, and follow-up records were obtained from the hospital's database. The SIRI was defined as follows: SIRI = Neutrophil (N) × Monocyte (M)/Lymphocyte (L), where N, M, and L are the pretreatment peripheral neutrophil, monocyte, and lymphocyte counts, respectively. To investigate the prognostic impact of SIRI, we determined a cut-off value for SIRI, which was obtained from the study group and was calculated by the receiver operating characteristics (ROC) curve analysis. The choice of threshold based on the Youden index (sensitivity + specificity − 1) was used to estimate sensitivity and specificity. The cohort of the study was analyzed in two groups according to the determined cut-off of SIRI.

The primary outcome of this study was to evaluate if SIRI has a diagnostic value for rectal cancer.

The secondary outcomes were to evaluate if SIRI is an independent prognostic factor for rectal cancer’s Overall Survival and the effect of SIRI on postoperative complications.

This study was approved by the Ethics Committee of Marmara University (numbered 05.05.2023.676) and it was performed in accordance with the ethical standards as laid down in the 1964 Declaration of Helsinki and its later amendments or comparable ethical standards, and informed consent was obtained from all patients.

### Statistical analysis

SPSS version 24.0 (Spss inc. IBM, Chicago, US) was used for statistical analysis. The data were presented as mean ± standard deviation (SD), median, and interquartile range (IQR). The proportion or frequency was compared between the two groups using Fisher’s exact test or the χ^2^ test, and differences in continuous variables were evaluated using the Student’s T-test and the Mann–Whitney U test for non-parametric values. Survival curves were compared using the Kaplan–Meier method and compared using the log-rank test.

## Results

**Fig. 1:**
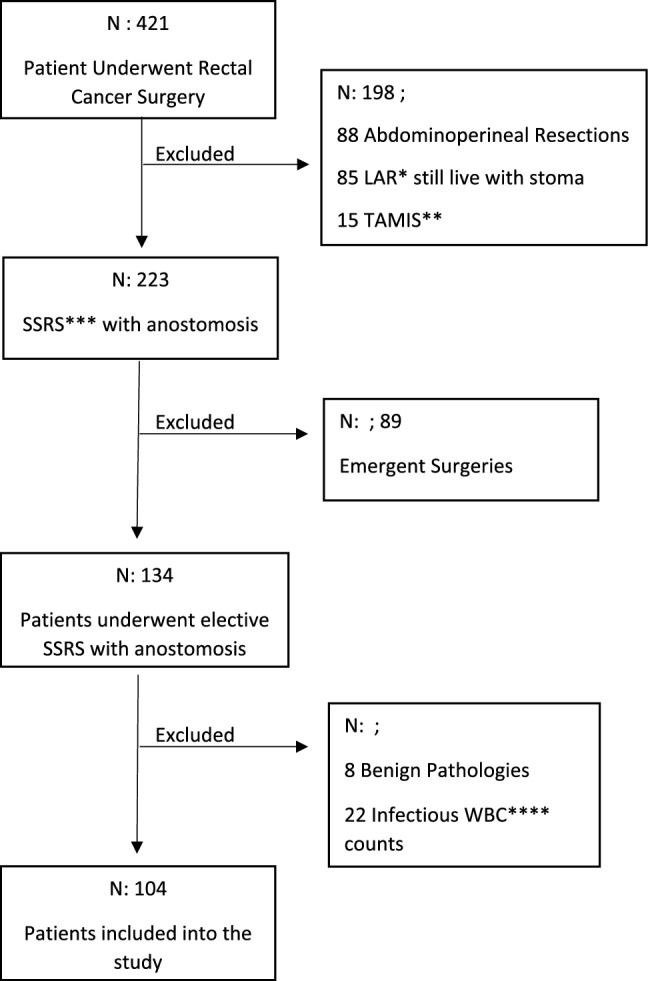
Patient Flowchart *Low Anterior Resection, **Transanal Minimal Invasive Surgery, *** Sphincter-Sparing Rectal Surgery, **** White Blood Cell

Between Jan./2017–Dec./2022, 421 patients underwent rectal cancer surgery in our center. Among them, 198 were excluded from the study group because they have APR, Transanal Minimal Invasive Surgery (TAMIS), underwent SSRS with a temporary diverting stoma and underwent a Hartmann procedure. Eighty-nine patients were emergent procedures. Of the remaining 134 patients with SSRS, eight patients had benign pathologies and were excluded. Twenty-two patients with suspicious infectious WBC counts were excluded. Finally, 104 patients were included in the study group (Fig. 1). A total number of 99 patients who underwent elective surgery for reasons other than cancer were included in the study as a control group.

**Fig. 2:**
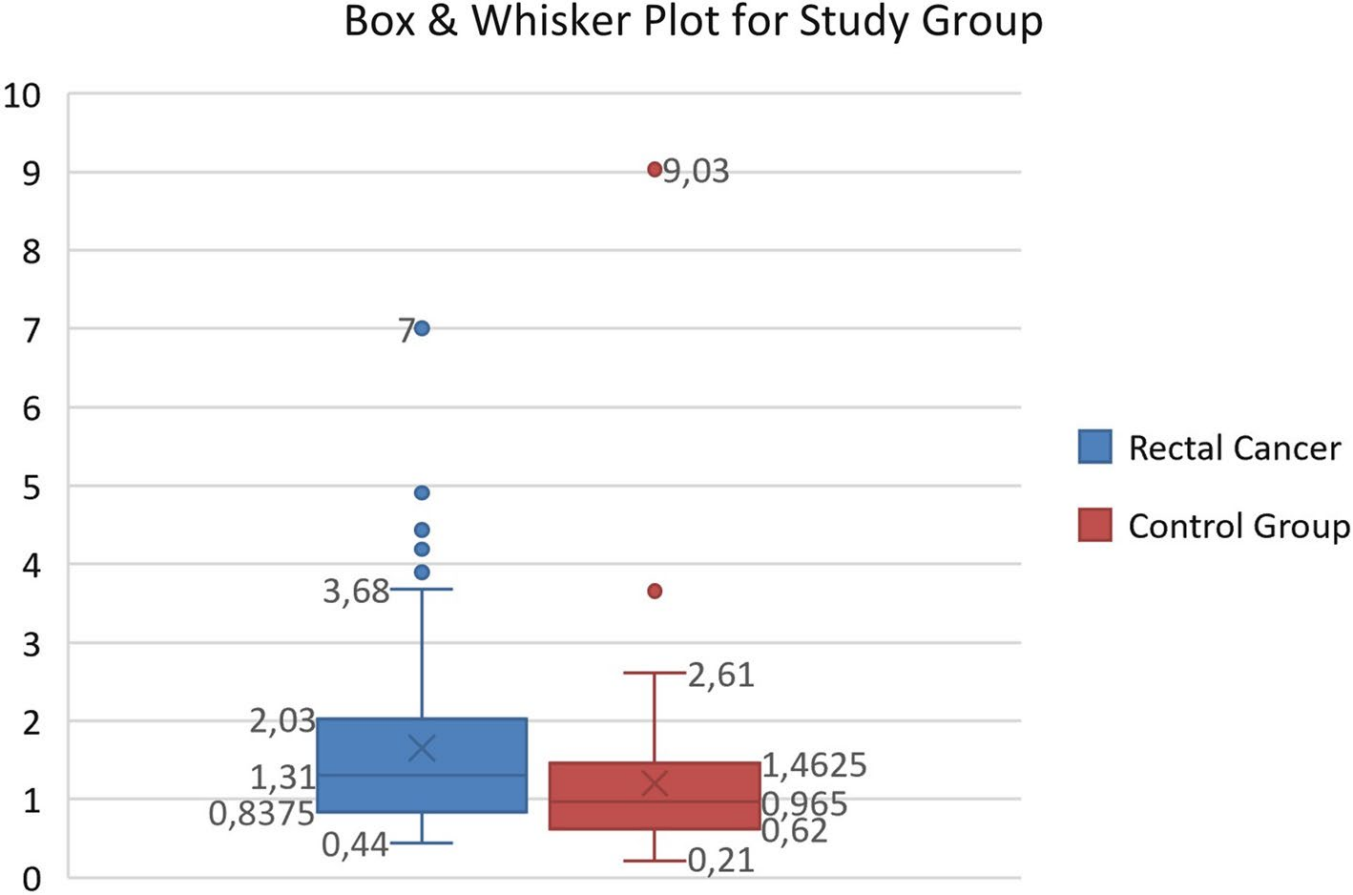
Box and whisker plot for rectal cancer and control group

The median SIRI value in the study group was significantly higher compared with the control group [respectively, 1.3 (IQR: 1.2) vs. 0.97 (IQR: 0.88), p: 0.003]. A box & whisker plot was used to demonstrate the distribution of SIRI levels between the two groups (Fig. 2). Whereas demographics between the rectal cancer group and the control group were not significantly different (Table 1).

**Table 1:** Basic characteristics between the study (rectum carcinoma patients) and the control group

N: 225	Study (N: 104)	Control (N:99)	p
Age (years) (mean ± SD)	62 (± 10.1)	60 (± 6.8)	0.099
Gender (%)			0.162
Male	59 (58%)	65 (66%)	
Female	45 (42%)	34 (34%)	
BMI (kg/m^2^) (mean ± SD)	26.7 (± 3.9)	27.5(± 4.3)	0.186
WBC* (10^9^ /L) (mean ± SD)	6.7(± 1.8)	6.9(± 1.5)	0.296
Neutrophil (10^9^ /L)	3.9(1.7)	4(1.8)	0.211
Monocyte (10^9^ /L)	0.5(0.3)	0.5(0.2)	0.567
Lymphocyte (10^9^ /L)	1.7(0.9)	2(0.8)	0.952
Platelet (10^9^ /L)	261(88)	238(80)	0.055
SIRI	1.3(1.2)	0.97(0.88)	*0.003
SIRI < 1.38			
SIRI $\le$ 1.38	56 (56%)	71 (72%)	*0.006
SIRI > 1.38	48 (44%)	28 (28%)	

*BMI* Body Mass Index, *WBC* White Blood Cell

*Bold emphasis reflects significant values

Our patient population comprised 59 (57%) male patients with a mean age of 62.09 ± 11 years. The majority of patients [64 (62%)] have one or more comorbid diseases. There were 27 (26%) patients who received neoadjuvant therapy and 55 (53%) patients with adjuvant therapy. The median follow-up time of the entire cohort was 33 (IQR: 43.5) months.

**Fig. 3:**
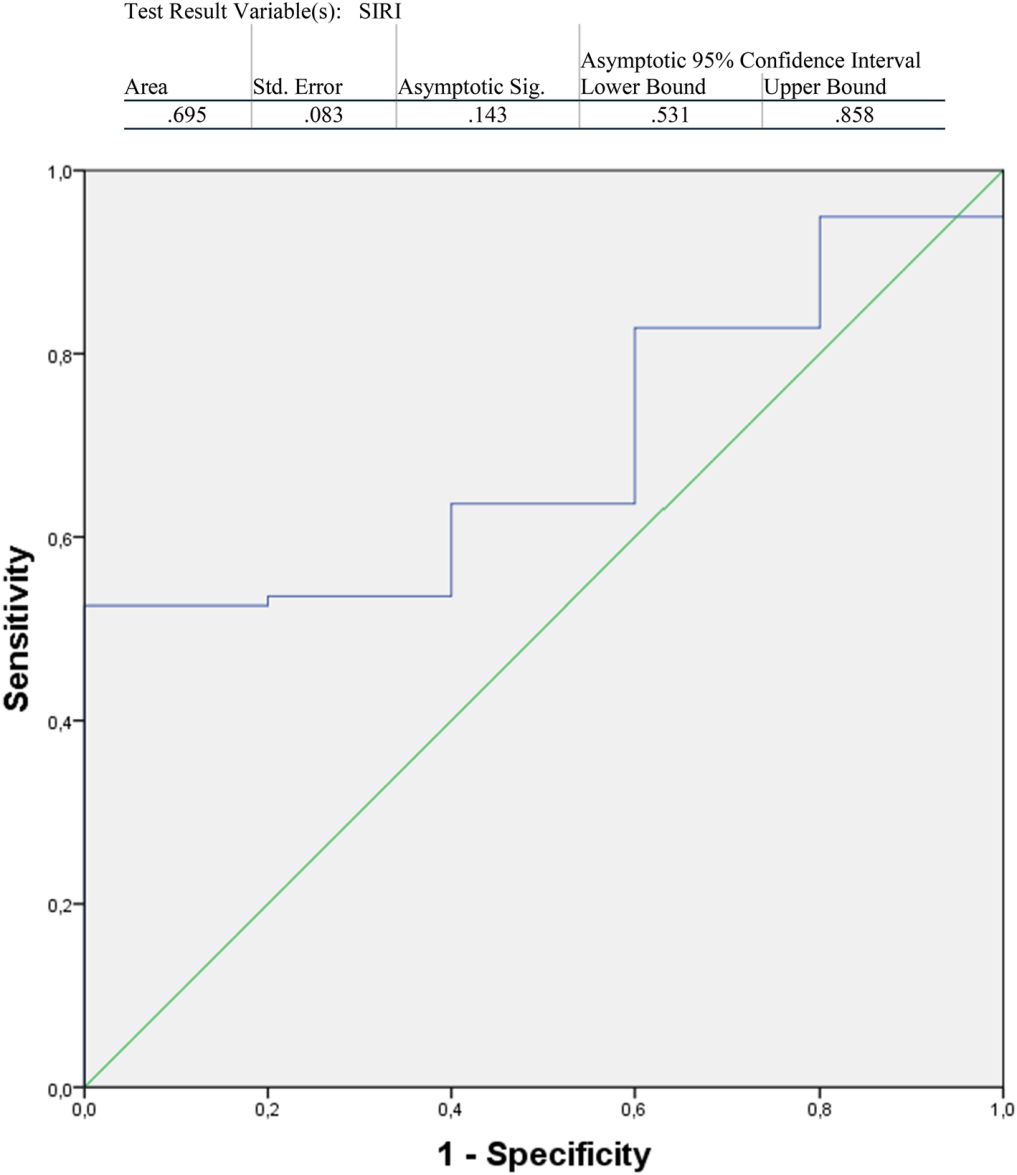
ROC curve analysis for SIRI

The ROC curve of SIRI regarding Overall Survival (OS) is shown in Fig. 3. Preoperative SIRI’s best cut-off value for predicting OS was estimated to be 1.38. Thus, the patients in the rectal cancer group were separated into two subgroups: SIRI ≤ 1.38 and SIRI > 1.38. There were 56 patients in the SIRI ≤ 1.38 group and 48 in the SIRI > 1.38 group. The patient demographics of the two rectal cancer groups are summarized in Table 2. TNM stages were similar between the two groups (Table 3).

**Table 2:** Patient Demographics in both groups

N: 104	SIRI $\le$ 1.38 (n: 56)	SIRI > 1.38 (n: 48)	p
Age (years) (mean ± SD)	61.39 (± 10.6)	62 (± 9.7)	0.901
Gender (%)			*0.002
Male	24 (43%)	35 (73%)	
Female	32 (57%)	13 (27%)	
BMI (kg/m^2^) (mean ± SD)	26.8 (± 4.)	26.4(± 3.8)	0.590
Comorbidities (%)			0.320
Presence	32 (57%)	32 (65%)	
Absence	24 (43%)	16 (35%)	
Neoadjuvant therapy	13 (23%)	14 (29%)	0.490
Adjuvant therapy	35 (62%)	20 (42%)	0.054
Hospital Stay(days) (mean ± SD)	4.8(± 1.6)	6.1(± 3.5)	*0.011
Hemoglobin* (g/dL) (median-IQR)	12.5(2.7)	13.6(2.9)	0.65
Albumin** (g/dL) (median-IQR)	4.2(0.4)	4.2(0.5)	0.717
Operation type			0.814
AR	7	5	
LAR	44	40	
VLAR	5	3	

*BMI* Body Mass Index, *AR* anterior resection, *LAR* low anterior resection, *VLAR* very low anterior resection

*Bold emphasis reflects significant values

**Table 3:** Pathological outcomes in both groups

N: 104	SIRI $\le$ 1.38 (n: 56)	SIRI > 1.38 (n: 48)	p
Stage T			0.411
Full-response	3	2	
T1	2	4	
T2	6	9	
T3	32	25	
T4	13	6	
Stage N			0.238
Full-response	2	2	
N0	28	30	
N1	20	13	
N2	8	3	
Stage M			0.551
M0	52	44	
M1	4	2	
Pathological stage			0.103
Full-response	2	2	
Stage I	6	11	
Stage II	14	16	
Stage III	30	15	
Stage IV	4	2	

Postoperative complications were observed to be significantly more frequent in the high SIRI group compared with the low SIRI group [respectively, 8 (11%) and 17 (31%), p: 0.004]. Postoperative complications were demonstrated in detail in Table 4. In both groups, there was no early postoperative mortality.

**Table 4:** Postoperative Complications in both groups

N: 104	SIRI $\le$ 1.38 (n: 56)	SIRI > 1.38 (n: 48)	p
Complication	(n: 5)	(n: 15)	*0.004
Wound infection	1	4	
Respiratory failure	0	1	
Facial dehiscence	0	1	
Anastomotic leak	1	3	
Ileus	3	6	
Complication grade^a^			
IIIA	1	3	
IIIB	3	5	
IVA	0	1	
IVB	0	0	
V	0	0	
≥ IIIA	4	9	0.074

* Bold emphasis reflects significant values

^a^Grade according to Clavian–Dindo classification

**Fig. 4:**
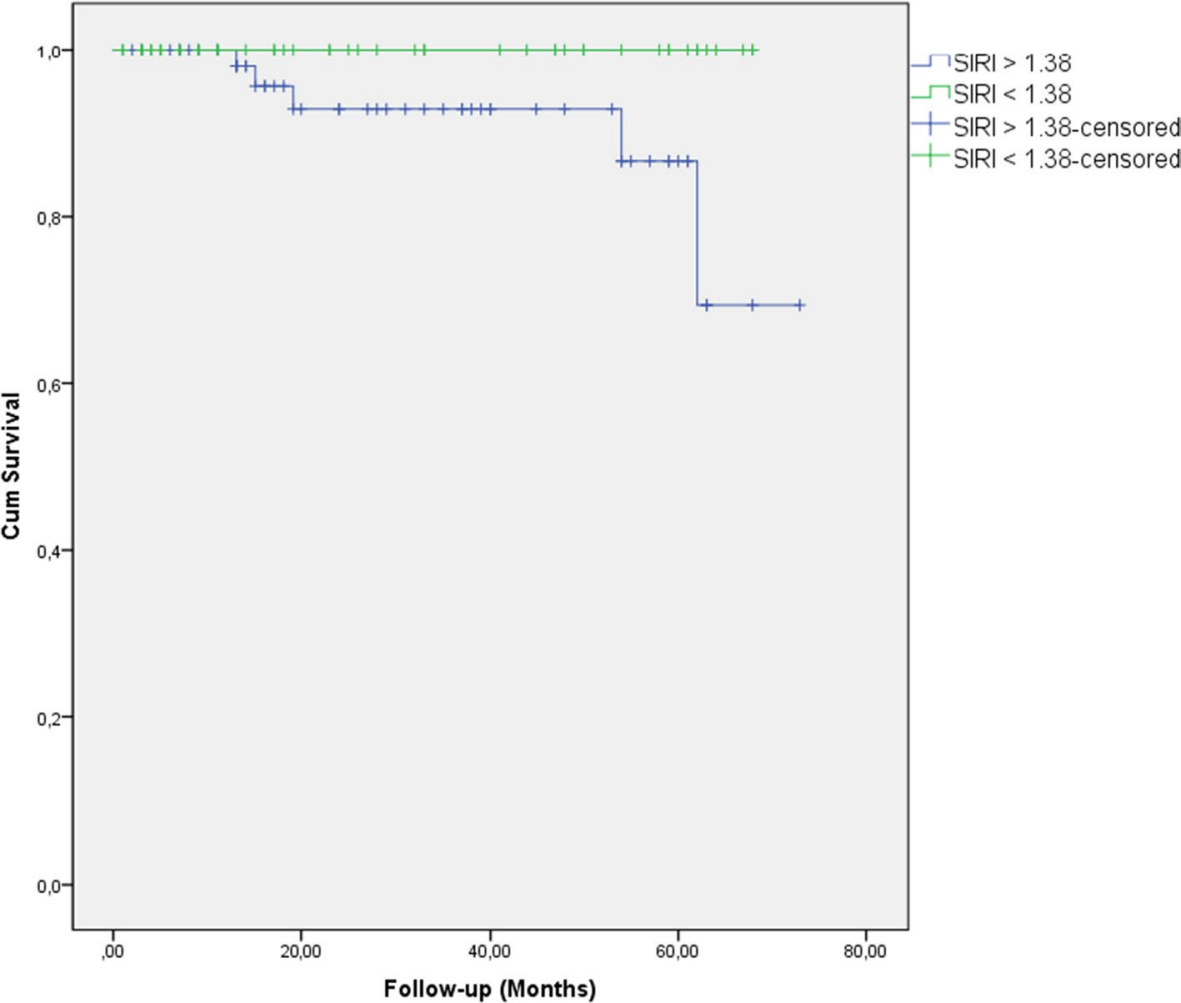
The association between the SIRI and Overall Survival (log-rank: 0.037)

The five-year overall survival (OS) of the rectal cancer group was 95%. The OS was significantly higher in the low SIRI group compared with the high SIRI group (respectively, 98% vs. 92%,lag-rank p: 0.037) (Fig. 4). However, in the multivariate analysis, SIRI was not an independent factor for the Rectal Cancer patient’s prognosis. Stage N was the only independent prognostic factor for rectal cancer in our study group (Table 5).

**Table 5:** Univariate and multivariate analysis for prognosis of rectal cancer

N:104	HR	95% CI	p	HR	95% CI	p
Gender	0.384	0.043–3.447	0.393			
Age	1.099	1.014–1.199	**0.021**	1.075	0.997–1.160	0.061
Neoadjuvant	1.706	0.065–5,283	0.634			
Adjuvant	0.665	0.089–4.949	0.690			
Comorbidity	0.625	0.067–5.826	0.680			
Pre-Operative Hb	0.703	0.446–1.108	0.129			
Operation Type	1,362	0,343–4.126	0.788			
Albumin	0.130	0.008–2.004	0.144			
Stage T (T1-T2 vs. T3-T4)	0.573	0.064–5.164	0.615			
Stage N (N0 vs. N+)	0.356	0.039–3.238	**0.003**	0.685	0.139–2.613	*0.035
Complication	0.726	0.477–1.107	0.137			
SIRI ≤ 1.38	1.804	0.424–2.802	**0.014**	0.894	0.406	1.802

*Bold emphasis reflects significant values

## Discussion

In this study, we investigated the diagnostic value prognostic impact of preoperative SIRI in patients with rectal cancer who underwent sphincter-sparing rectal surgery (SSRS). The median SIRI value in the study group was significantly higher compared with the control group. The overall survival (OS) was significantly higher in the low SIRI group compared with the high SIRI group. Furthermore, Postoperative complications were observed to be significantly more frequent in the high SIRI group compared with the low SIRI group.

Although few studies investigated the relationship between SIRI and colorectal cancers, to our knowledge, no studies investigated the diagnostic value of SIRI in cancer patients and the prognostic impact of SIRI in rectal cancer patients. The exact underlying mechanism of the close correlation between the Inflammatory markers and poor prognosis is still not clear. Neutrophils may enhance metastasis, operate as reservoirs for circulating vascular endothelial growth factor, and directly interact with circulating cancer cells. Typically, lymphocytes play a crucial role in tumor suppression by causing cytotoxic cell death and releasing cytokines that prevent the growth and metastasis of cancer cells. The presence of lymphocytes in the tumor may be a favorable prognostic sign [15]. Nevertheless, memory T cells in colorectal cancer can change the tumor matrix or tumor cells in the adaptive immune response to reduce the metastatic potential of tumor cells. It may be that the transport characteristics, density, and long-term anti-tumor ability of T-cells play a central role in controlling tumor recurrence [16]. Similarly, tumor-infiltrating lymphocytes in solid tumors exhibit oligoclonal proliferation, tumor antigen recognition, and tumor-specific cytolytic activity in vitro, contributing to improved clinical outcomes such as delayed recurrence and mortality. Therefore, elevated SIRI, caused by lymphocytopenia and/or a high neutrophil count, may lead to a poor immune response to malignancy and an increased potential for tumor recurrence. Moreover, these ratios might have a potential during the diagnosis period of malignancies. Many studies discuss the prognostic performance of these indexes. However, there are only a few reports regarding the diagnostic value of inflammatory markers. Abbate et al. recently reported that SIRI might have a strong diagnostic performance on salivatory gland tumors [17]. Finally, evidence in this report may lead following studies on diagnostic performances of inflammatory markers. This is the first study to compare the SIRI between cancer patients and the control group.

There are a few limitations of this study. The first is its retrospective design. Second, there is a small number of patients included in the analysis. Lastly, the lack of power analysis is considered one of the limitations of this study.

Recent research mostly focused on the prognostic impact of SIRI on various cancers. In contrast, there are only a few studies designed to find the diagnostic and predictive value of SIRI. Moreover, these primarily concentrate on conditions other than cancer. Liu et al. found that high SIRI levels were associated with an increased risk of lower extremity deep venous thrombosis [18]. Urbanowicz et al. reported that SIRI levels higher than 1.22 had a higher probability of single and complex coronary disease [19]. Committeri et al. used inflammatory markers to differentiate Warthin tumors from pleomorphic adenoma and malignant neoplasms [20]. They reported SIRI with an accuracy of 0.83 and significant differences in the univariate Kruskal–Wallis test. Nevertheless, studies that investigated SIRI’s diagnostic value on cancer are still missing. Therefore, our case–control study might fill this gap.

Systemic inflammation affects the local tumor microenvironment and is crucial for the growth and metastasis of cancer [21]. Growing evidence has indicated the predictive value of inflammation-related variables in rectal cancer patients with various baseline characteristics and TNM stages [22]. Different inflammatory markers, such as NLR, PLR, and Lymphocyte to Monocyte Ratio (LMR), have been used to predict prognosis in rectal cancer patients [23]. In addition, SIRI was recently used to determine OS in many kinds of cancers [24]. However, to our best knowledge, this is the first study that evaluates the prognostic significance of SIRI in RC patients.

There are a few studies that have discussed the effects of SIRI on colorectal cancer patient’s prognosis. Cao et al. evaluated the correlation of SIRI with tumor-associated bacteria and the prognosis of colorectal cancer patients [25]. They reported that SIRI had a superior predictive performance than the other inflammation-related markers, such as NLR, PLR, and LMR. Cai et al. found a positive correlation between high levels of SIRI and poor prognosis in colorectal cancer patients [16]. In their cohort, SIRI and MLR showed the strongest relationship with their outcome events in a median 23-month follow-up. However, this study focused more on developing a novel marker. Thus, the univariate and multivariate analysis of existing markers was insufficient.

Especially in the last two decades, treatment of RC differed from the remaining colon cancers. Recently, preoperative NCRT has taken a growing role in the treatment of RC. Hence, the results of inflammation-related markers need to be reevaluated for RC. Nagasaki et al. included 202 rectal cancer patients received neoadjuvant chemo-radiotherapy (NCRT) in their study [26]. They reported that NLR has an impact on the OS of RC patients with NCRT; however, they found no significant correlation with recurrence-free survival. In another cohort, Shen et al. found similar results to the previous study. They analyzed 199 patients underwent rectal surgery after NCRT. NLR was significantly associated with poor OS. On the contrary, disease-free survival has no significant relation with NLR. These two studies used different cut-off values for NLR (NLR ≤ 5.0 vs. NLR ≤ 2.8, respectively). The optimal cut-off values for every inflammation-related marker remain controversial.

Systemic chemotherapy might have a suppressive role in systemic inflammation, and might affect the peripheral inflammatory cells [27]. Formica et al. argued that a decrease in the NLR after chemotherapy in metastatic colorectal cancer patients may reflect poor prognosis [28]. In other words, lower NLR showed a significant correlation with OS in metastatic colorectal patients. Preoperative NCRT has been taking a substantial role in RC treatment. Therefore, the results and predictive performance of SIRI might be affected. Further investigations are needed on this topic.

Several studies showed that systemic inflammation is effective in demonstrating postoperative complications. Sugimoto et al. reported that Naples prognostic score, which developed from inflammatory biomarkers, was an independent predictive marker for severe postoperative complications [29]. Tong et al. showed that increased NLR and serum eotaxin-2 levels were strongly related to postoperative complications [30]. In our cohort, high SIRI levels were associated with higher complication rates, and this was consistent with the existing literature.

## Conclusion

Rectal cancer patients have higher SIRI levels than the control group, which can be added to other diagnostic tools and could be a diagnostic marker in rectal cancer patients. Higher SIRI levels were also associated with poorer prognosis and increased complication rates. Still, further prospective randomized studies with a large number of patients are needed.

## Data Availability

The datasets used and/or analyzed during the current study are available from the corresponding author upon reasonable request.
